# Circulating Microparticles in the Pathogenesis and Early Anticoagulation of Thrombosis in COVID-19 With Kidney Injury

**DOI:** 10.3389/fcell.2021.784505

**Published:** 2022-01-18

**Authors:** Chengyue Wang, Chengyuan Yu, Valerie A. Novakovic, Rujuan Xie, Jialan Shi

**Affiliations:** ^1^ Department of Hematology, The First Hospital of Harbin, Harbin Medical University, Harbin, China; ^2^ Department of Nephrology, The First Hospital of Harbin, Harbin Medical University, Harbin, China; ^3^ Department of Geriatric, Shenzhen People’s Hospital (The Second Clinical Medical College, Jinan University, The First Affiliated Hospital, Southern University of Science and Technology), Shenzhen, China; ^4^ Department of Research, VA Boston Healthcare System, Harvard Medical School, Boston, MA, United States; ^5^ Department of Medical Oncology, Dana-Farber Cancer Institute, Harvard Medical School, Boston, MA, United States

**Keywords:** COVID-19, microparticle, kidney, thrombosis, phosphatidylserine, early anticoagulation

## Abstract

As more is learned about the pathophysiological mechanisms of COVID-19, systemic thrombosis has been recognized as being associated with more severe clinical manifestations, mortality and sequelae. As many as 40% of patients admitted to the hospital due to COVID-19 have acute kidney injury, with coagulation abnormalities the main cause of impaired function. However, the mechanism of renal thrombosis and the process leading to kidney injury are unclear. Microparticles (MPs) are membrane bubbles released in response to activation, injury or apoptosis of cells. The phosphatidylserine (PS) exposed on the surface of MPs provides binding sites for endogenous and exogenous FXase complexes and prothrombin complexes, thus providing a platform for the coagulation cascade reaction and facilitating clot formation. In the context of COVID-19 infection, viral attack leads immune cells to release cytokines that damage circulating blood cells and vascular endothelial cells, resulting in increased MPs levels. Therefore, MPs can be used as a risk factor to predict renal microthrombosis and kidney injury. In this paper, we have summarized the latest data on the pathophysiological mechanism and treatment of renal thrombosis caused by MPs in COVID-19, revealing that the coagulation abnormality caused by MP and PS storms is a universal progression that aggravates the mortality and sequelae of COVID-19 and potentially other pandemic diseases. This paper also describes the risk factors affecting renal thrombosis in COVID-19 from the perspective of the Virchow’s triad: blood hypercoagulability, vascular endothelial injury, and decreased blood flow velocity. In summary, given the serious consequences of thrombosis, current guidelines and clinical studies suggest that early prophylactic anticoagulant therapy reduces mortality and improves clinical outcomes. Early anticoagulation, through inhibition of PS-mediated coagulopathy, allows maintenance of unobstructed blood circulation and oxygen delivery thereby facilitating the removal of inflammatory factors, viruses, MPs, and dead or damaged cells, and expediting patient rehabilitation.

## Introduction

As many as 40% of patients admitted to the hospital due to COVID-19 have acute kidney injury (AKI) (Blum, et al., 2020), and these patients have more serious clinical manifestations and mortality (Zheng, et al., 2020; Chebotareva, et al., 2021). Virus invasion, complement activation, and local inflammation can directly damage the kidneys. However the kidneys are more commonly damaged by hypercoagulability, systemic microvascular disease and thromboembolism. Many studies have shown that the pathophysiology of SARS-CoV-2 infection involves haemostatic imbalance. Importantly, microthrombi form in the early stages of COVID-19 infection and circulate through the blood to the lungs, kidney, heart, brain, liver, spleen, and intestinal arteries, worsening the disease (Helms, et al., 2020; Nicolai, et al., 2020). Immunity thrombotic regulation is a key indicator of COVID-19 severity. The results of pathology and imaging suggest that systemic microvascular disease including the kidneys is accompanied by microthrombosis (Chen and Pan, 2021; Idilman, et al., 2021). Excessive activation of coagulation can induce acute lung injury, respiratory dysfunction, disseminated intravascular coagulation, multiple organ failure and even death by inducing thrombotic complications, excessive inflammation and tissue damage (Fei, et al., 2020). Therefore, thrombosis is not a bystander of COVID-19, but a central pathogenic factor that connects respiratory failure and systemic hypercoagulability and multiple organ dysfunctions including kidneys (Nicolai, et al., 2020). Extracellular vesicles (EVs) include exosomes and microparticles (MPs), are thought to be important coagulants because their exposed phosphatidylserine (PS) provides binding sites for endogenous and exogenous FXase complexes and prothrombinase complexes, thereby promoting coagulation (Tripisciano, et al., 2017; Fendl, et al., 2018). In addition, our team has reported that MPs and PS exposure is an important coagulant factor in COVID-19 and its related complications, sepsis, inflammatory bowel disease and other kidney diseases (Gao, et al., 2012; He, et al., 2016; Ma, et al., 2017; Yu, et al., 2018; Chen, et al., 2020; Liu et al., 2021a; Zhang, et al., 2021). In COVID-19, two studies have demonstrated the procoagulant effect of EVs and MPs (Balbi, et al., 2021; Zahran, et al., 2021). It can be seen that MPs and PS lead to microthrombi and macrovascular thrombosis in COVID-19 patients.

There have been reports showing long-term effects of COVID-19 on the kidneys, with some patients experiencing AKI recurrence, chronic kidney injury (CKD), post-injury inflammation, and fibrosis (Yende and Parikh, 2021). Although this phenomenon cannot be clearly explained at present, vascular damage and thrombosis caused by persistent viruses and inflammation may lead to secondary damage to the kidneys. Therefore further study of the thrombosis mechanism in COVID-19 could lead to better management of complications such as kidney injury and delay the progression of kidney disease. In this article, we first introduce the basic process of MPs release and PS exposure as a mechanism which promotes thrombosis. Second, we explain the risk factors affecting renal thrombosis in COVID-19 from the perspective of the Virchow’s triad: blood hypercoagulability, vascular endothelial injury, and decreased blood flow velocity. Through this framing we explain in detail that vascular endothelial disease is the core mechanism of COVID-19 lung-to-kidney thrombosis. SARS-CoV-2 destroys circulating blood cells, renal capillary endothelial cells and podocytes, releasing great amounts of PS^+^ MPs, leading to the production of thrombin, increased blood viscosity, and promoting renal thrombosis. Third, we summarize and analyze the current guidelines and studies, showing that early prophylactic anticoagulation can prevent renal and systemic thrombosis, improve clinical outcomes, reduce mortality and sequelae, and promote early recovery of patients.

## The Source of MPS and the Mechanism Promoting Thrombosis

EVs are sub-cellular structures released by most eukaryotic cell types and include exosomes and MPs. They can mediate the exchange of a broad array of molecules between adjacent or distant cells by transferring their cargo (proteins, RNA, lipids, and carbohydrates, etc), along with membrane receptors and antigen presentation complexes. As a result, they have become recognized as an important medium for intercellular communication. Over the past years, the critical role of EVs in hemostasis and thrombosis has been described (Chatterjee, et al., 2020; Tripisciano, et al., 2020). In general, MPs, but not exosomes, have thrombotic activity. During the process of apoptosis and activation of many cell types, such as platelets, epithelial cells, endothelial cells, dendritic cells, B cells, T cells, mast cells, and tumor cells, MPs are secreted under the influence of cytokines, thrombin, endotoxins, physical stimuli or hypoxia. Platelets are the source of up to 70% of MPs, which can play a role in various disease pathologies (Che, et al., 2021). In addition, glomerular podocytes have been shown to release MPs in response to hypoxia, high glucose, and inflammatory stimuli, contributing to disease progression (Sharma, 2020; Takahashi, et al., 2020). Normally, the outer leaves of the plasma membrane are rich in lipids such as phosphatidylcholine and sphingomyelin, while the inner leaves contain PS and phosphatidylethanolamine. This asymmetry is maintained by the synergistic activity of flippase, floppase, and scramblase. Flippase and floppase depend on ATP to flip PS into the inner and outer membrane, respectively. Scramblases mediate bidirectional transport of lipids through Ca^2+^ signaling. Cell activation or injury leads to increased Ca^2+^ influx, flippase and floppase occlusion, and scramblase activation (Guo, et al., 2020; Nagata, et al., 2020). This results in the shedding of PS-exposing MPs which can be detected through binding of lactadherin or annexin V (Xie, et al., 2012). PS^+^ MPs promote clotting by providing binding sites for both endogenous and exogenous FXase complexes and prothrombin complexes. MPs also bind to cells through specific adhesion receptors that stimulate production of tissue factors (TF) and cytokines that promote clotting, inflammation, and endothelial dysfunction.

In summary, thrombosis can be caused by any PS exposure on either MPs or activated, damaged, or dying cells [including white blood cells (monocytes, neutrophils, lymphocytes), platelets, red blood cells (RBCs), ECs or smooth muscle cells (SMCs)]. This activation can be precipitated by conditions such as: cancer, trauma, surgery, or infection. In the various inflammatory diseases listed in Table 1 (diabetic nephropathy, nephrotic syndrome, uremia, coronary heart disease, antiphospholipid antibodies, dengue, COVID-19), live or damaged cells (including RBCs, platelets, lymphocytes, monocytes, granulocytes, and ECs) all release a large amount of PS^+^ MPs (Suades, et al., 2012; Gao, et al., 2012; Gao, et al., 2015; Jy, et al., 2015; Chaturvedi, et al., 2015; He, et al., 2016; Patil, et al., 2018; Yu, et al., 2018; Zahran, et al., 2021). It should be noted that although the study by Zahran et al. indicated that circulating PMPs and EMPs are the main sources of MPs in COVID-19, MPs generated from other cells may also play a role. In COVID-19, the virus directly damages cells and leads to PS^+^ MPs release. It can also indirectly activate the immune system (monocytes, neutrophils, lymphocytes), produce a cytokine storm, and result in a large amount of cell damage and death. Importantly, the destruction of vascular ECs by SARS-CoV-2 and immune inflammation is the central link in the formation of thrombosis (Gu, et al., 2021), which inevitably leads to a greater proportion of EMPs. The proportion of various types of cell MPs in the circulating blood of COVID-19 patients warrants further study.

**TABLE 1:** The main source of microparticles in various diseases.

Disease	References	RMPs	PMPs	EMPs	NMPs	LMPs	MMPs
DKD	Yu, M. et al	↑	↑	↑	↑	↑	↑
NS	Gao, C. et al	↑	↑	↑	↑	↑	↑
Uremia	Gao, C. et al	↑	↑	↑	↑	↑	↑
IBD	He, Z. et al	↑	↑	↑	↑	↑	↑
CABG	Jy, W. et al	↑	↑	↑	↑	↑	↑
Atherothrombosis	Suades, R. et al	↑	↑	↑	↑	↑	↑
APS	Chaturvedi, S. et al	↑	↑	↑	↑	↑	↑
Dengue	Patil, R. et al	↑	↑	↑	↑	↑	↑
COVID-19	Zahran, A. M. et al	↑	↑	↑	↑	↑	↑

DKD, diabetic kidney disease; NS, nephrotic syndrome; IBD, inflammatory bowel disease; CABG, coronary artery bypass graft; APS, antiphospholipid syndrome; MPs, microparticles; RMPs, RBC MPs; PMPs, platelet MPs; EMPs, endothelial cell MPs; NMPs, neutrophils MPs; LMPs, leukocyte MPs; MMPs, monocytes MPs.

Blue shading indicates the main sources of MPs found in the study.

## Release of MPS, Renal Thrombosis and Renal Injury in COVID-19

**FIGURE 1:**
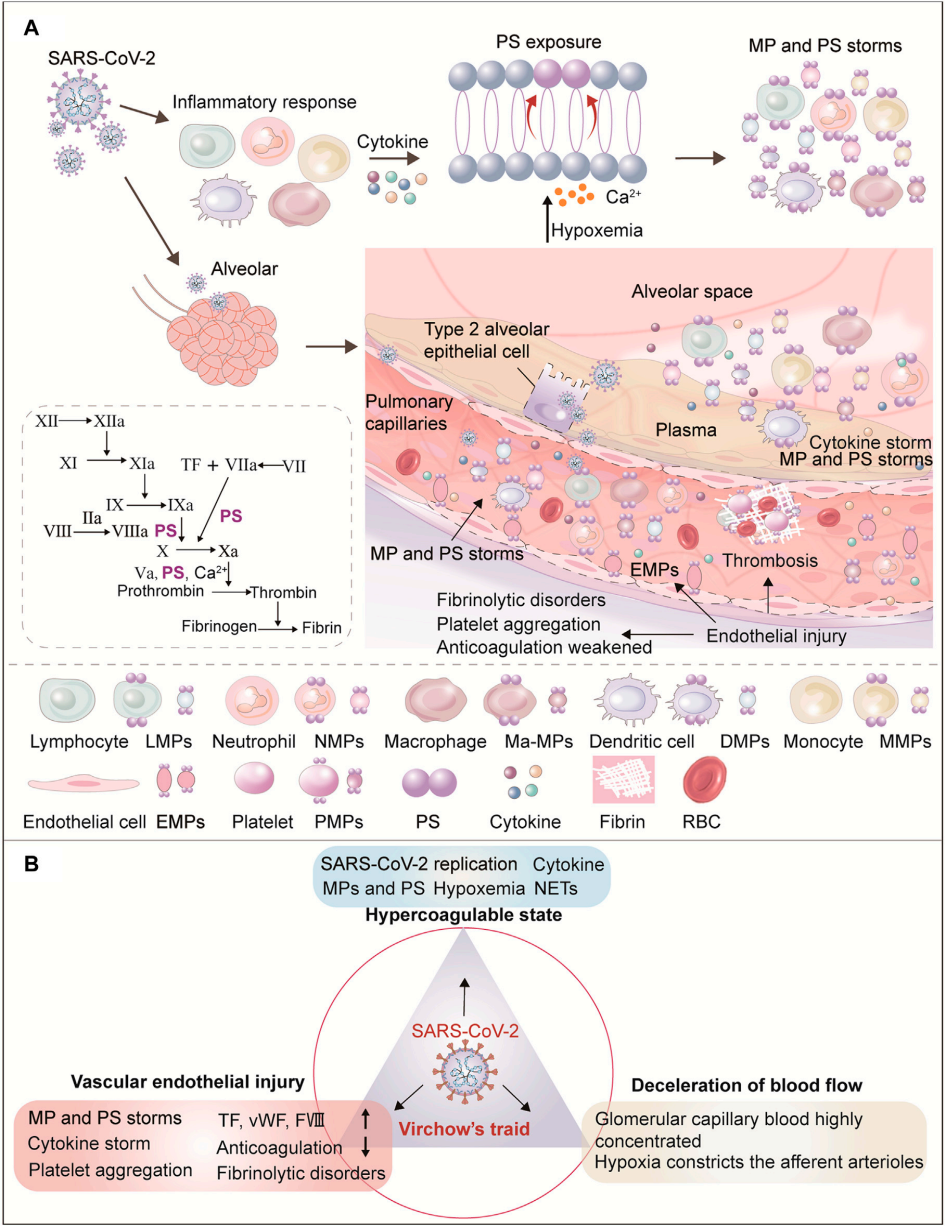
Role of circulating microparticles in the pathogenesis of thrombosis in COVID-19. **(A)** This figure shows the mechanism of MPs production in COVID-19. During the invasion and replication of SARS-CoV-2, the body first initiates immune cell recruitment (i.e., monocytes, macrophages, dendritic cells, neutrophils, and T cells) and the release of cytokines. After cell activation and injury, ATP production is reduced and consumption increases. With the resulting increase in intracellular Ca^2+^, two ATP-dependent transposases (flippase and floppase) are blocked, and ATP-independent scramblases are activated. This leads to the exposure of phosphatidylserine (PS) in the outer cell membrane, accompanied by the shedding of microparticles (MPs). SARS-CoV-2 replicates in alveolar epithelial cells, damages the membrane and releases the virus, infecting more adjacent alveolar epithelial II cells and pulmonary capillary endothelial cells (ECs), resulting in vascular endothelial injury. As the main triggering site of clot formation, vascular endothelial function and integrity destruction can lead to endothelial MPs release, up-regulation of TF expression on cell surfaces, platelets activation, increased vWF and FVIII, and thus promote platelet aggregation and thrombosis. The damaged endothelium is shed, weakening the normal endothelial anticoagulation functions and causing fibrinolysis disorder. Finally, alveolar capillary microthrombosis results in insufficient blood perfusion, leading to hypoxemia. In conclusion, increased viral load, hypoxemia, endothelial injury, and inflammatory response jointly promote PS and MP storms which drive the coagulation cascade. **(B)** Diagram of renal thrombosis in COVID-19 from the perspective of Virchow’s triad. Virchow’s triad composes abnormal blood composition (hypercoagulable blood state), vascular endothelial damage, and decreased blood flow velocity. The three elements of Virchow are themselves mutually influenced and inseparable. Of the three, systemic vascular endothelial injury is the main triggering factor for COVID-19 clot formation. Endothelial dysfunction caused by viruses, hypoxia, immune response and hypercoagulable state, platelet activation, high viscosity and abnormal blood flow lead to thrombosis in COVID-19.

**FIGURE 2:**
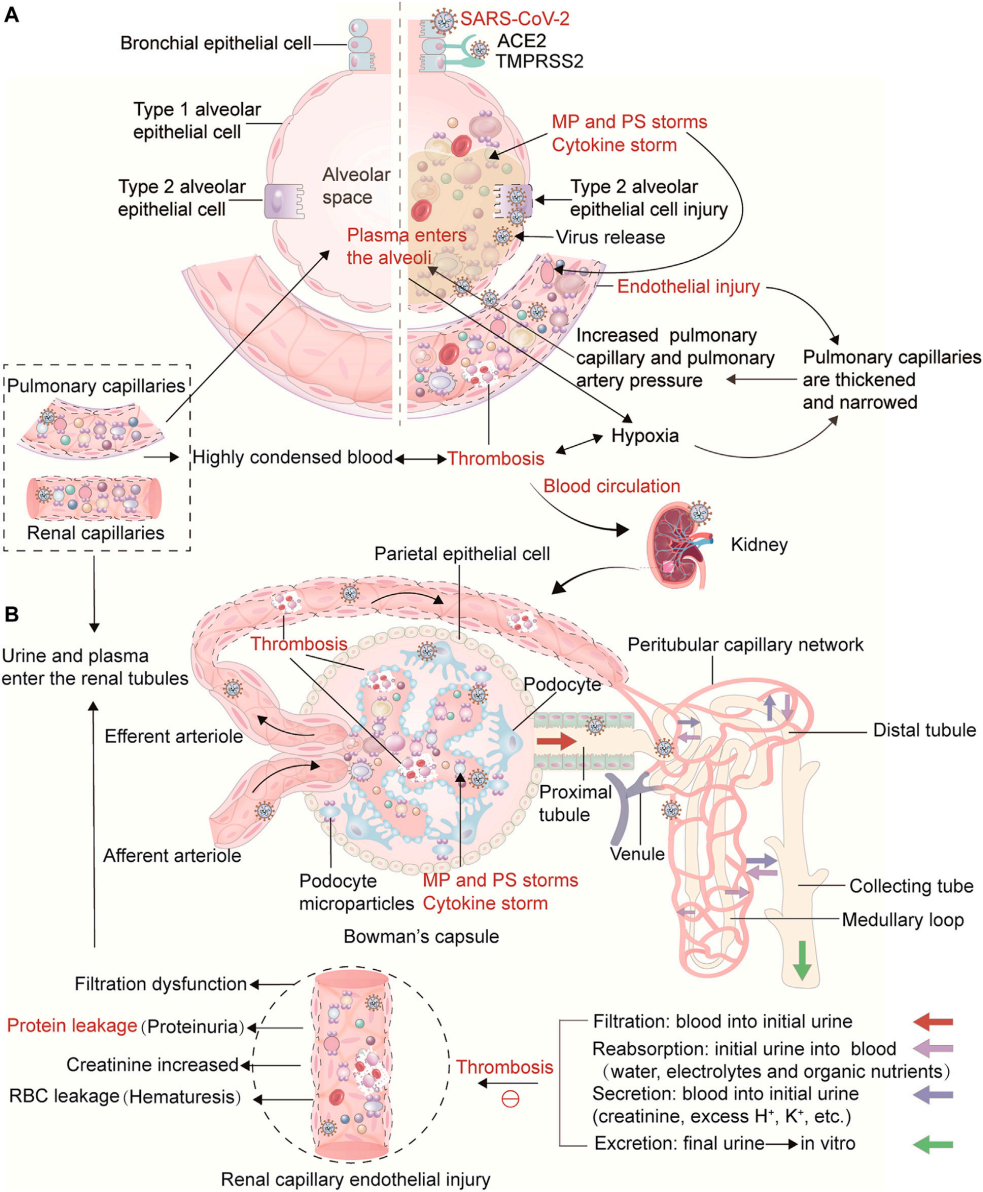
Thrombosis from the lung to the kidney in COVID-19 and its influence on the physiological process of renal hemofiltration and initial urine production. Illustration of the link between thrombosis in the lungs and the kidneys in COVID-19. **(A)** In the lungs, viruses, cytokines, PS^+^ MPs and hypoxia lead to damage and contraction of pulmonary capillary endothelial cells, thickening and narrowing of capillaries, and ultimately causing pulmonary hypertension. As the pressure differential increases, plasma and a small number of erythrocytes in the pulmonary capillaries are pushed into the alveolar space, disrupting air-blood exchange. As plasma is forced into the alveoli, the water is evaporated by respiration, leaving tremelloid proteins. The patients develop progressive dyspnea as the air space in the alveoli decreases. Ultimately, thrombosis can result in increased metabolic toxicants, energy deficiency, massive cell damage and death, and multiple organ failure. As the disease progresses, SARS-CoV-2 can travel through the bloodstream from infected alveolar type II epithelial cells and pulmonary capillaries ECs to distant blood vessels, including the glomerular filtration barrier ECs and podocytes, gradually invading various organs. **(B)** This part represents the physiological process of kidney hemofiltration and initial urine production. When blood flows into the glomerulus through the afferent arterioles, it is filtered through the glomerular filtration barrier and capillary walls. The filtered water and soluble substances enter the renal capsule, and then the filtrate enters tubules to form initial urine. The capillaries around the renal tubules are connected with the filtrate in the tubules to ensure the reabsorption of water, electrolytes and organic nutrients, and waste products such as creatinine, excess H^+^ and K^+^ are actively excreted in the filtrate. Due to the high pressure in the glomerulus, the blood in the glomerular capillaries is highly concentrated, the hematocrit increases, and the fluidity is significantly reduced. Simultaneously, the blood in the glomerular capillaries is highly concentrated, the hematocrit increases, and the fluidity decreases significantly. Importantly, SARS-CoV-2, cytokines, and hypoxia damage glomerular capillary endothelial cells and podocytes, affect their metabolism, lead to insufficient ATP production, activate scramblase, release PS^+^ MPs, and promote thrombin production, which hinders blood circulation, increasing blood viscosity and kidney thrombosis. In addition, renal tubular epithelial cells are damaged, resulting in renal filtration dysfunction. This further activates the blood coagulation pathway and the accumulation of procoagulant substances, which hinders the flow of concentrated blood, and ultimately causes the glomerular filtration rate, renal tubular reabsorption, secretion and excretion functions to decrease, leading to symptoms such as increased creatinine, hematuria, and proteinuria.

SARS-CoV-2 is a β coronavirus with a single stranded RNA genome. It can penetrate into ciliary cells of nasal mucosa, larynx, trachea, bronchus, bronchioles and gradually into the terminal respiratory bronchioles by binding to angiotensin-converting enzyme 2 (ACE2) and transmembrane protease receptor serine protease 2 (TMPRSS2) receptor targeting cells. Existing studies have shown that the expression of ACE2 and TMPRSS2 is widely distributed in human tissues such as lung, liver, kidney, heart, brain and many digestive organs. In addition, ACE2 and TMPRSS2 are highly expressed in renal tubule cells, suggesting that SARS-CoV-2 may directly bind renal ACE2-positive cells and destroy renal tubule function (Li et al., 2021; Dong et al., 2021). During the invasion and replication of SARS-CoV-2, the body first initiates the innate and acquired immune responses, causing the recruitment of monocytes, macrophages, dendritic cells, neutrophils and T cells, releasing cytokines (Renu et al., 2021; Kaur et al., 2021) and triggering MPs release and PS exposure. As COVID-19 progresses, the virus invades and replicates in crescent alveolar epithelial cells, while previously infected pulmonary capillary ECs aggravate vascular endothelial injury (Guglielmetti et al., 2020; Perico et al., 2021), recruit inflammatory factors, and produce MP and PS storms. As the main triggering site of thrombosis, vascular endothelial disfunction can lead to the release of endothelial MPs, up-regulation of TF expression on the cell surface, and platelets activation, which promotes platelet aggregation and thrombosis (Gu, et al., 2021). The damaged endothelium is shed, causing fibrinolysis disorder and disruption of the normal endothelial anticoagulation function (Figure 1A). Meanwhile, alveolar capillary microthrombi result in insufficient blood perfusion, leading to hypoxemia. Hypoxia, in turn, results in the contraction of pulmonary capillary ECs which thickens and narrows the capillaries, ultimately causing pulmonary hypertension. As the pressure differential increases, plasma and a small number of erythrocytes in the pulmonary capillaries are pushed into the alveolar space, disrupting air-blood exchange and aggravating respiratory failure. As plasma is forced into the alveoli, the water is evaporated by respiration, leaving tremelloid proteins (Figure 2A). The patients develop progressive dyspnea as the air space in the alveoli decreases. Importantly, hypoxemia can result in increased metabolic toxicants, energy deficiency, massive cell damage, and multiple organ failure. Moreover, production of EVs increases under hypoxic conditions (Almeria et al., 2019). As the disease progresses, SARS-CoV-2 can travel through the bloodstream from infected alveolar type II epithelial cells and pulmonary capillaries ECs to distant blood vessels, including the glomerular filtration barrier ECs and podocytes, gradually invading various organs.

The classic Virchow’s triad explains the risk factors associated with thrombosis: abnormal blood composition (hypercoagulable blood state), vascular endothelial damage, and decreased blood flow velocity (Wolberg et al., 2012; Ding et al., 2020). The three elements of Virchow are themselves mutually influenced and inseparable. ECs regulate vasomotion and secrete anticoagulant substances, and changes in blood flow velocity and blood composition also affect vascular endothelial integrity, function, and vascular reactivity. Similarly, Virchow’s triad can be used to explain thrombosis associated with COVID-19 (Figure 1B). Looking at renal blood flow, the physiological process of renal hemofiltration, when blood flows into glomerulus through the afferent arterioles, initial urine is formed through continuous plasma filtration (Scott and Quaggin 2015). The capillaries around the renal tubules contact the filtrate in the tubules to ensure the reabsorption of water, electrolytes and organic nutrients, while waste products such as creatinine, excess H^+^ and K^+^ are actively excreted into the filtrate (Gueutin et al., 2012). Since the diameter of efferent arterioles is smaller than that of the afferent arterioles, the blood flow through the glomerulus is more resistant, and the capillary pressure is higher than the capillary pressure in other parts of the body. As the blood in the glomerular capillaries is highly concentrated, the hematocrit increases, and the fluidity decreases significantly. The concentrated blood finally enters the capillary network of the proximal tubule through the glomerular efferent arterioles. Only after the reabsorption of water by the proximal tubule do the RBC specific volume and blood flow gradually recover. Therefore, reduced blood flow in the glomerular capillary network and the proximal tubule capillary network lead to an increased risk of thrombotic coagulation and vascular blockage. Importantly, circulating SARS-CoV-2 and inflammatory factors lead to damage and contraction of the glomerulus and peritubular capillary endothelium, and infection of renal tubular epithelial cells (Ahmadian et al., 2021; AndradeSilva et al., 2021), causing renal filtration dysfunction. This further activates the coagulation pathway and the accumulation of activated coagulation factors, hinders blood circulation, and results in RBC aggregation. Eventually, the glomerular filtration rate, renal tubular reabsorption, secretion and excretion function decreases, leading to symptoms such as increased creatinine, hematuria, and proteinuria (Figure 2B).

Next, we focus on blood hypercoagulability and endothelial injury. As mentioned earlier, systemic vascular endothelial injury is the main triggering factor for COVID-19 clot formation. By directly attacking and stimulating cytokines, SARS-CoV-2 damages circulating blood cells, renal capillary ECs and podocytes, which affects their metabolism, decreases ATP production, weakens sodium pump activity, leading to cell edema. A large amount of Ca^2+^ enters the cells and activates scramblase, causing PS exposure and the release of PS^+^ MPs. These PS-rich membranes promote thrombin production, thus slowing blood flow, increasing blood viscosity and renal thrombosis. In addition, hypoxia can aggravate renal afferent arteriole contraction, renal capillary perfusion insufficiency, increase acid, and promote contraction deformation of ECs, impairing endothelial barrier function and converting ECs into a pro-coagulation and pro-inflammatory phenotype (Chen et al., 2020). In addition, a recent study described the existence of smoldering inflammation in COVID-19 (Schmidt 2021). Continued attacks from viruses and cytokines will aggravate endothelial damage and hinder endothelial repair. In conclusion, viruses, cytokines, and PS^+^ MPs jointly participate in COVID-19 thrombosis from lungs to kidneys and other organs. The clotting will damage the vascular endothelium, causing a vicious circle, and ultimately leading to the decline of respiratory function and kidney filtration (Figure 2).

After renal thrombosis, the glomerular capillary endothelium, renal tubular epithelial cells and podocytes are damaged. Reduced renal blood flow and abnormal blood distribution are the main mechanisms of acute renal insufficiency with decreased glomerular filtration rate and oliguria. Furthermore, renal ischemia and hypoxia can also aggravate renal tubular damage, resulting in reabsorption and secretion dysfunction. In the late phase of COVID-19, some patients may develop septic shock, followed by hypotension and vasoconstriction that can worsen acute tubular necrosis. Especially in the process of renal ischemia-reperfusion injury, inflammatory factors and reactive oxygen species produced by renal tubular epithelial cells and renal parenchymal cells can again cause the accumulation of inflammatory cells such as neutrophils. This releases more MPs and inflammatory mediators, resulting in a vicious cycle and exacerbating thrombosis and kidney injury (Hesp et al., 2020; Dadson et al., 2020).

Studies have analyzed the impact of chronic kidney failure (CKD) on COVID-19 progression and mortality (Leon-Abarca et al., 2020). CKD patients often have comorbidities such as hypertension, cardiovascular disease and diabetes. It is clear that these patients have poor quality of life, slow recovery and poor prognosis. This kind of chronic disease itself can lead to the destruction of nephrons, chronic inflammation and hypoxia caused by long-term hyperlipemia and hyperglycemia that will aggravate the damage to kidney capillaries and endothelium, leaving the patient in a state of hypercoagulation (Addi et al., 2018). Acute SARS-CoV-2 infection results in not only renal and systemic thrombosis, but also significant increases in ICU events, mechanical ventilation, disability, and mortality (Nadim et al., 2020; Yende and Parikh 2021).

## Clinical and Laboratory Findings of Kidney Thrombosis in COVID-19

In the early stages of COVID-19, patients often have no renal manifestations, though laboratory tests can show hematuria and mild proteinuria. Later aggravation of kidney injury results in increased creatinine and blood urea nitrogen in blood, decreased glomerular filtration, and significantly lower lymphocytes, albumin and hemoglobin (Chan et al., 2020; Ke et al., 2021). Abnormal coagulation indicators are also common in COVID-19 patients, including increased D-dimer, prolonged PT and aPTT, and thrombocytopenia (Smarz-Widelska et al., 2021). At the vascular level, acute kidney injury images showed renal vasoconstriction, increased vascular permeability, and microthrombosis. In the advanced stages of the disease, CT may be helpful in diagnosing renal thromboembolism. A recent case report found renal infarction in two patients with COVID-19 and evidence of extrarenal thromboembolism in one of them. Renal infarction resulted in loss of kidney function in both patients (Post et al., 2020). Another study, involving 31 patients, found lung and kidney hypoperfusion in a subset of patients with mild to moderate COVID-19 on dual-energy computed tomography (DECT) scans, which may indicate systemic microangiopathy with microthrombi (Idilman et al., 2021). It has an impact on the management of COVID-19 patients, such as early indications of thrombosis prevention or anticoagulant drugs and optimization of oxygenation strategies. In addition, autopsy observations of the kidneys of COVID-19 patients revealed diffuse and focal segmental fibrin thrombus in the glomerular capillary loops and endothelial injury in the glomeruli (Cheng et al., 2020; Smarz-Widelska et al., 2021). Injury to the renal proximal tubules is also a common manifestation found at autopsy (Lax, et al., 2020).

Most early AKI was persistent, and the severity and duration of AKI were clearly related to all aspects of the underlying COVID-19 disease process (Wan et al., 2021). Many patients return to normal levels of serum creatinine after AKI, but the kidneys may not fully recover. A study showed that increased severity of the acute infection (regardless of whether the patient is ambulatory, hospitalized, or intensive care), was correlated to a graded increase in the risk of ongoing kidney disease (Bowe et al., 2021). In order to avoid the occurrence of kidney damage, many experiments were devoted to the study of effective methods for early identification and prediction of kidney damage (Khokhar et al., 2020; Liu et al., 2021b). Among them, the detection of clinically classified potential risk factors, procalcitonin and eGFR helps clinicians to identify patients with kidney injury early (Liu et al., 2021a). Early diagnosis of COVID-19-related AKI and initiation of treatment, including adequate hemodynamic support, treatment management, and avoidance of renal viral drugs may help improve the patient’s prognosis (Gabarre, et al., 2020).

## Treatment

Based on the above analysis of the pathophysiological mechanisms of renal thrombosis and kidney injury, we assert that anticoagulation treatment of COVID-19 is particularly important. Vascular damage can be sustained by both direct damage to the vascular wall and systemic inflammation. Blood clots further aggravate vascular disease and cause secondary damage to the organs they supply (Schmidt 2021). The kidney is a common target for the virus. In addition to the early proteinuria, blood urine and kidney function checks, it is crucial to identify patients with renal function changes, so preventive anticoagulation can be started as soon as possible. Early therapy to prevent the activation of blood coagulation factors (virus, cytokines, MPs, injury and dead cells) to prevent blood clots can control disease progression and reduce the morbidity and mortality. Furthermore, since hypoxia can occur in patients in the early stages of COVID-19, oxygen should be provided as early as possible, even if blood oxygen saturation is normal. Oxygen can delay the occurrence of hypoxemia, ensure normal cell metabolism, and hinder patient deterioration.

### Anticoagulant Therapy

In view of the serious consequences of thrombosis, anticoagulation has become the core of comprehensive management of COVID-19 (Pretorius et al., 2021). Most current guidelines support anticoagulant prophylaxis for hospitalized patients with COVID-19 (Spyropoulos et al., 2020; Moores et al., 2020; Orsi et al., 2020; Vanassche et al., 2020; Gerotziafas et al., 2020), but the optimal timing and dosage of anticoagulant are still controversial. There have been no separate studies to observe the efficacy of anticoagulants in the treatment of renal thrombosis. Regarding the application of anticoagulants, the most common one is low-molecular-weight heparin (LMWH). In addition to its anticoagulant effects, heparin can also bind to cytokines, protect ECs, inhibit chemotaxis, and control leukocyte migration and complement activation (Gozzo et al., 2020; Buijsers et al., 2020). Because renal dysfunction reduces the clearance rate of LMWH, prolonging its half-life, which leads to a higher risk of bleeding, unfractionated heparin (UFH) should be used preferentially in patients with renal insufficiency. Recent guidelines refer to the use of DOACs in COVID-19 outpatient and discharge patients to prevent arterial and venous thrombosis (Spyropoulos et al., 2020; Moores et al., 2020). However, all DOACs are cleared by the kidney, and patients with renal insufficiency may be at risk of delayed clearance and bleeding. In conclusion, anticoagulants should not be used in COVID-19 patients with kidney injury if they are primarily cleared through the renal system.

The mortality from kidney injury in patients with severe COVID-19 is significantly higher than that in patients with mild-to-moderate COVID-19, because of the increased prevalence of clots in patients with advanced COVID-19. Thus, it has been suggested that LMWH should be increased to moderate or therapeutic doses in late stages (Table 2). However, the effect of increasing the dosage of anticoagulant is not ideal. A study showed that in critically ill COVID-19 patients, compared with usual-care pharmacologic thromboprophylaxis, therapeutic-dose anticoagulation with heparin did not lead to greater probability of survival to hospital discharge or a greater number of days free of cardiovascular or respiratory organ support (REMAP-CAP Investigators et al., 2021a). Another study showed that intermediate-dose prophylactic anticoagulation did not improve major comprehensive outcomes, including all-cause mortality and venous thromboembolism, compared with standard-dose prophylactic anticoagulation in ICU patients (Inspiration Investigators et al., 2021). Rates of major bleeding were higher in the intermediate-dose group than in the standard-dose group (2.5 vs. 1.4%). In fact, as the disease progresses, the incidence of thromboembolism in COVID-19 patients is gradually increasing. Coagulation dysfunction, inability to move, systemic inflammation, mechanical ventilation, and central venous catheters are all factors that aggravate thrombosis and poor prognosis (Cui et al., 2020; Blasi et al., 2020). Therefore, the application of anticoagulant therapy in severe COVID-19 pneumonia is necessary, but the above studies have shown that the beneficial results of anticoagulation did not depend on increasing the dose. Late-stage thrombosis is associated with high consumption of coagulation factors and cannot be affected by high doses of heparin (TenCate 2021). In addition, larger anticoagulant dosages increase the risk of bleeding. Severe patients often present with multiple underlying diseases, a history of hormone therapy or serious liver and kidney dysfunction risk factors and therefore cannot avoid the increase incidence of bleeding.

**TABLE 2:** Trial design, population, interventions, and outcomes for COVID-19 inpatients undergoing anticoagulation.

Studies	Population	Intervention	Results	Conclusions
Rentsch C. T et, al. (February 2021)	*N* = 4,297, Non-anticoagulant (*n* = 670), Anticoagulant (*n* = 3,627)	Prophylactic anticoagulation	30-day mortality in anticoagulant and non-anticoagulant patients (14.3 vs. 18.7%)	Early initiation of prophylactic anticoagulation compared with no anticoagulation among patients admitted to hospital with COVID-19 was associated with a decreased risk of 30-day mortality and no increased risk of serious bleeding events
Sadeghipour P. et al. (April 2021)	*N* = 562 (ICU) Intermediate-dose (*n* = 276), Standard prophylactic anticoagulation (*n* = 286)	Intermediate-dose: enoxaparin, 1 mg/kg daily Standard prophylactic: enoxaparin, 40 mg daily	Massive bleeding rate in medium dose group and standard dose group (2.5 vs. 1.4%)	These results do not support the routine empirical use of intermediate-dose prophylactic anticoagulation in unselected patients admitted to the ICU with COVID-19
Arslan Y et, al. (June 2021)	*N* = 413 LMWH-treated patients (*N* = 187), LMWH-untreated patients (*N* = 226)	Early anticoagulant (LMWH)	The number of ICU transfer and longer length of hospital stay were more commonly observed in LMWH-untreated patients (pvalues <0.05)	Early anticoagulant treatment with relatively higher doses of LMWH may improve the clinical outcome of COVID-19 patients and shorten the length of hospital stay
Lopes R. D et, al. (June 2021)	*N* = 615 Therapeutic group (*n* = 311), Prophylactic group (*n* = 304)	Therapeutic: rivaroxaban for stable patients, or enoxaparin or unfractionated heparin for unstable patients, followed by rivaroxaban to day 30, Prophylactic: standard in-hospital enoxaparin or unfractionated heparin	The primary efficacy outcome was not different between patients assigned therapeutic or prophylactic anticoagulation, Therapeutic vs. prophylactic: bleeding (8 vs. 2%)	In-hospital therapeutic anticoagulation with rivaroxaban or enoxaparin followed by rivaroxaban to day 30 did not improve clinical outcomes and increased bleeding compared with prophylactic anticoagulation
Goligher E. C et, al. (August 2021)	*N* = 1,098 (critical patients) Therapeutic-dose (*n* = 534), Usual-care thromboprophylaxis (*n* = 564)	Therapeutic-dose anticoagulant (heparin)	Therapeutic-dose vs. usual-care: median value for organ support (1 vs. 4); survival discharge rate (62.7 vs. 64.5%); major bleeding (3.8 vs. 2.3%)	In critically ill patients with COVID-19, therapeutic-dose anticoagulation with heparin did not result in a greater probability of survival to hospital discharge or a greater number of days free of cardiovascular or respiratory organ support than did usual-care pharmacologic thromboprophylaxis
Lawler P. R et, al. (August 2021)	*N* = 2,219 (noncritical patients)	Therapeutic-dose anticoagulant (heparin)	Therapeutic-dose anticoagulation increased organ support-free days was 98.6%, Therapeutic-dose vs. usual-care thromboprophylaxis: major bleeding (1.9 vs. 0.9%)	In noncritically ill patients with COVID-19, an initial strategy of therapeutic-dose anticoagulation with heparin increased the probability of survival to hospital discharge compared with usual-care thromboprophylaxis

A higher anticoagulant dose seems to have some beneficial effects in intermediate stage patients. One study showed that therapeutic doses of heparin reduced the use of cardiovascular or respiratory organ support, increased the probability of survival in hospital and after discharge, but also increased rates of major bleeding compared with patients receiving thromboprophylaxis (1.9 vs. 0.9%) (REMAP-CAP Investigators et al., 2021b). Another large clinical study of all hospitalizations, including those in the middle and late stages, showed therapeutic anticoagulation did not improve clinical outcomes and increased bleeding compared with prophylactic anticoagulation (Lopes et al., 2021). So, the question is whether an early anticoagulation is advantageous? The early-stage patients do not have severe thrombocytopenia and consumption of coagulation factors, and pro-coagulant substances such as cytokines and PS^+^ MPs have not yet been released in large quantities. Can the occurrence of bleeding events be avoided without increasing the anticoagulant dose? There have been articles supporting early prophylactic anticoagulation (Terpos et al., 2020; Rentsch et al., 2021; Arslan et al., 2021; Schulman and Harenberg 2021; Kollias et al., 2021). One study showed that early initiation of prophylactic anticoagulation was associated with a reduced risk of 30-day death without an increased risk of serious bleeding events (Rentsch et al., 2021). Another study showed that early LMWH anticoagulation improved clinical outcome and reduced the length of hospital stay (Arslan et al., 2021). Combined with the pathophysiological mechanisms of COVID-19 thrombosis, thrombogenesis in severe COVID-19 is driven by cytokine storms, MP and PS storms. Microthrombosis can lead to more diffuse perfusion injury and extensive dysfunction of the affected organs (Bray et al., 2020). This also means that in early mild patients, prophylactic anticoagulation may maintain unobstructed blood flow and oxygen delivery, remove inflammatory factors, and promote monocytes and macrophages to clear viruses and blood cells damaged and dead by virus attacks (Merad and Martin 2020). It prevents the virus from contacting and destroying ECs and inhibits the procoagulant platform provided by MPs and PS storms, thereby inhibiting thrombosis. In addition, cells (such as monocytes, macrophages, etc.) present antigens in the process of clearing the virus, stimulate the production of antibodies, promote the early recovery of patients, avoid the progression of the disease to the middle or late stages, and greatly reduce mortality and sequelae. This may helps explain the benefits of anticoagulation prophylaxis in early-stage patients.

### Continuous Renal Replacement Therapy (CRRT)

SARS, MERS and septicemia have been successfully treated with sustained CRRT in the past. CRRT can support the kidney to maintain the stability of the internal environment of the body, and remove various pathogenic substances such as inflammatory mediators, cytokines and uremic toxins. Successful uses of CRRT in patients with severe COVID-19 have been reported with significant remission (Benedetti et al., 2020; Ronco et al., 2020). However, clinical trials are needed to determine the effectiveness and safety of the strategy.

## Conclusion

Knowing pathophysiological role of MPs in COVID-19 renal thrombosis has enhanced our understanding of the relationship between viruses and coagulation and will help formulate effective anticoagulation strategies. Although renal injury and poor prognosis are multifactorial, renal thrombosis plays an extremely important role. The Virchow’s triad highlights the relationship between blood hypercoagulability, endothelial injury and blood flow velocity. It is also reasonable to analyze the extent to which renal thrombosis is caused by inflammation, MP and PS storms, vascular endothelial injury and renal hemodynamic changes in COVID-19. In addition, coagulation abnormalities caused by MP and PS storms are a universal feature that aggravates the mortality and sequelae of pandemic diseases such as COVID-19 and other acute inflammatory diseases. As we all know, there is currently no effective drug for SARS-CoV-2. The effectiveness of antiviral drugs has been controversial and immunosuppressants have destructive effects on autoantibody production. As the virus mutates, the effective life of the vaccine is uncertain, and new vaccines will need to be constantly developed. In future epidemics similar to SARS, MERS and COVID-19, we should not ignore the coagulation abnormalities caused by the release of MPs and PS exposure, and pay attention to the application of anticoagulation therapy.

## Abbreviations

COVID-19, Coronavirus disease 2019; SARS-CoV-2, severe acute respiratory syndrome coronavirus 2; EVs, extracellular vesicles; MPs, microparticles; PS, phosphatidylserine; RBC, red blood cells; ECs, endothelial cells; ACE2, angiotensin-converting-enzyme 2; TMPRSS2, transmembrane protease receptor serinase 2; CKD, chronic kidney failure; PT, prothrombin time; APTT, activated partial thromboplastin time; AKI, acute kidney injury; LMWH, low-molecular-weight heparin; DOACs, direct oral anticoagulants; UFH, unfractionated heparin; CRRT, continuous renal replacement therapy.
